# A Fully Replicable Exercise Program for Individuals with Sleep-Disordered Breathing: Protocol Design and Training Load Monitoring

**DOI:** 10.3390/jfmk10030311

**Published:** 2025-08-12

**Authors:** Jose M. Saavedra, Katrin Y. Fridgeirsdottir, Conor J. Murphy, Harald Hrubos-Strøm, Erna S. Arnardottir

**Affiliations:** 1 Physical Activity, Physical Education, Sport and Health (PAPESH) Research Centre, Sports Science Department, School of Social Sciences, Reykjavik University, 101 Reykjavík, Iceland; katrinf@ru.is (K.Y.F.); conorm@ru.is (C.J.M.); 2 Reykjavik University Sleep Institute (RUSI), 101 Reykjavik, Iceland; ernasifa@ru.is; 3 Department of Otorhinolaryngology, Division of Surgery, Akershus University Hospital, 1478 Oslo, Norway; harald.hrubos-strom@medisin.uio.no; 4 Institute of Clinical Medicine, University of Oslo, Campus Akershus University Hospital, Clinic of Surgery, 1478 Lørenskog, Norway; 5 Department of Engineering, School of Technology, Reykjavik University, 101 Reykjavik, Iceland; 6 Department of Computer Science, School of Technology, Reykjavik University, 101 Reykjavík, Iceland

**Keywords:** circuit training, concurrent exercise, rating of perceived exertion, intensity, training load

## Abstract

**Objectives**. The objectives of this study were (i) to design in detail an exercise program for individuals with sleep-disordered breathing (SDB) that would be reproducible, and (ii) to present a system for monitoring training load (volume × intensity) within such a program. **Methods**. A comprehensive exercise program was developed for individuals with SDB, detailing not only the session structure (warm-up, main part—circuit training and brisk walking—and cool-down) but also the specific exercises, training volume (actual exercise time excluding rest), intensity (Borg Rating of Perceived Exertion—RPE), and training load (calculated as time × RPE, in arbitrary units). This detailed program was previously implemented in a RCT (ISRCTN16974764). A comparison was also made between the planned and performed intensity, and training load through a paired t-test. **Results**. A fully replicable program was presented. No significant difference was found between the planned and performed training load (*p* = 0.482). When analyzed by week, a significant difference was found only for overestimation in weeks 9–12 (*p* < 0.001). **Conclusions**. In general terms, it can be concluded that a detailed exercise program was described for individuals with SDB. The program is reproducible in terms of content, training volume, intensity, and load. Moreover, the RPE proved to be a valid parameter for quantifying intensity, allowing for the integration of all parts of the session, as well as various types of content. The planned and performed programs (as quantified via participants’ RPE) matched appropriately. Therefore, this program can be reproduced and applied to this type of population.

## 1. Introduction

Exercise is widely recognized as one of the most important health-promoting behaviors; there have even been initiatives that consider “exercise is medicine” [1]. Adults aged 18–64 years are recommended to engage in 150–300 min of moderate-intensity aerobic activity, or 75–150 min of vigorous-intensity aerobic activity per week, or an equivalent combination of both, complemented by muscle-strengthening activities, two or more days per week [2]. Meeting these recommendations has been shown to offer extensive health benefits [3] and reduce the risk of various chronic conditions, including cardiovascular disease [4], hypertension [5], type II diabetes [6], and certain cancers [7], and it has positive effects on sleep [8]. Additionally, exercise can help reduce symptoms of anxiety and depression [9] and enhance health-related quality of life. However, to achieve greater improvements across multiple parameters, programs must consider both the individual characteristics of participants and the specific features of the disease to ensure effectiveness and safety, while understanding each patient’s individual risk can help tailor treatment decisions, maximizing benefits and minimizing associated risks [10].

More recently, exercise programs have gained attention as a potential treatment option for sleep-disordered breathing (SDB), particularly obstructive sleep apnea (OSA) [11]. Although continuous positive airway pressure remains the gold-standard treatment for moderate to severe OSA [12], adherence issues and limited impact on comorbidities have underscored the need for adjunct or alternative therapeutic approaches [13]. Emerging evidence indicates that exercise interventions can reduce the severity of OSA as assessed by the apnea-hypopnea index (AHI) [14,15,16,17,18,19,20,21,22]. In addition to its effects on AHI, exercise can have a positive influence on other parameters depending on the type of exercise performed. Aerobic exercise can help reduce weight, improve cardiovascular and respiratory function, enhance metabolic health, reduce fluid retention in the rostral region, and promote better sleep quality. Resistance exercise is also beneficial for weight control and may support cardiovascular and metabolic health. Finally, mind–body exercises (such as Tai Chi or yoga) have a positive impact on overall sleep patterns and respiratory function [23].

To optimize the effectiveness of exercise programs, both in general and for SDB patients in particular, program design should adhere to some of the training principles [24,25]. The exercise program needs to challenge the body beyond its current capacity to stimulate physiological adaptations, referred to as overload [24]. This can be achieved by manipulating training variables such as volume intensity and frequency, or volume. Without sufficient overload, physiological systems will not be sufficiently stressed to generate improvements in cardiovascular fitness, muscular strength, or respiratory function, resulting in a progress plateau. However, this overload needs to follow a progression, a gradual increase in training stress over time, in response to improving fitness [25]. Progression helps to sustain adaptation while minimizing the risk of overtraining and injury, especially in individuals who are sedentary or living with chronic conditions [26].

In this way, to ensure that programs adhere to the principles of training, it is essential to describe and control volume (e.g., duration of the exercise session), intensity (e.g., level of exertion), frequency (e.g., exercise sessions per week), as well as training load (volume × intensity). While volume and frequency are often described, intensity can be measured in many ways. For instance, researchers have used various methods to monitor intensity during exercise interventions which can make comparisons challenging. Nonetheless, monitoring and controlling the intensity of an exercise intervention is a critical step [27] since, for example, different intensities may result in similar adaptations [28]. Intensity control is necessary to facilitate reproducibility and to help determine the degree of adaptation to exercise [29]. Intensity can be monitored using different methods [30]. One of these methods is heart rate (HR), which is often expressed as a percentage of maximum HR or HR reserve (minimum to maximum HR). It is widely used due to its non-invasive nature and its responsiveness to aerobic load. On the other hand, lactate threshold monitoring requires blood sampling, involves time delays, and is generally less practical during large-scale exercise interventions. Another method of quantifying intensity is the Borg Rating of Perceived Exertion (RPE) [31]. This method is not only used in the sports field [32], but also for exercise prescription in the health field [17,19]. The RPE scale ranges from 6 to 20 and is used to assess how hard a person feels they are working during physical activity or exercise. The scale starts at six, which represents no exertion at all, such as resting in a chair. From seven to eight, the effort is considered very, very light, and nine to ten is described as very light, typically how one feels during slow walking. Levels 11 to 12 are fairly light to somewhat hard, representing low to moderate intensity. The target range for exercise is usually between 13 and 16, with 13 to 14 being somewhat hard and 15 to 16 considered hard, reflecting a challenging but sustainable effort. Ratings of 17 to 18 are categorized as very hard, and 19 to 20 indicate extremely hard to maximal exertion, which should be avoided in general populations due to the intensity involved [31]. This RPE has shown a strong correlation with heart rate [33]. If intensity is not controlled, researchers risk drawing inaccurate conclusions that might be related to the intensity rather than the effectiveness of an exercise intervention. In clinical populations with chronic conditions, exercise intensity is even more complicated and crucial. Precise prescription is important not only for effective adaptations but also for participant safety [34].

In this context, the objectives of this study were (i) to design in detail an exercise program for individuals with sleep-disordered breathing that would be reproducible, and (ii) to present a system for monitoring training load (volume and intensity) within such a program.

## 2. Materials and Methods

### 2.1. Design

The exercise program presented is part of the intervention in the randomized controlled trial (RCT) registered under the number ISRCTN16974764. This RCT included two interventions (an exercise program and a lifestyle app intervention) compared to a control group. It analyzed the severity of SDB (AHI and snoring), anthropometry, body composition, physical fitness (handgrip strength and aerobic endurance), and health-related quality of life, among other parameters assessed at baseline and at the end of the 12-week intervention. The initial results of this RCT were recently published [16].

### 2.2. Participants and Procedures

One hundred ninety-two participants (age 37.4 ± 6.3 years, BMI 33.3 ± 4.2 kg·m^−2^, 52.6% males) were evaluated using a three-night self-applied polysomnography, anthropometric measurements (height, weight, body mass index, neck circumference), body composition (using a TANITA MC-780, Tokyo, Japan), physical fitness (handgrip dynamometry and six-minute walking test), and health-related quality of life (HRQoL) (SF-36 questionnaire). The severity of SDB, assessed based on the AHI (events/h; mean ± standard deviation) in each of the three groups (see Section 2.1) prior to the intervention, was as follows: exercise group, 17.7 ± 14.6; app group, 12.1 ± 9.7; control group, 14.9 ± 11.4, with no statistically significant differences between the three groups (F = 2.302; *p* = 0.104) [16]. The exercise program led to a reduction in AHI and improvements in skeletal muscle mass, physical fitness, and four domains of health-related quality of life [16]. All participants received information about the study’s objectives, and providing written informed consent was required for inclusion. The study was approved by the Icelandic Data Protection Authority and the National Bioethics Committee of Iceland (ref. no. 22-082), and it was conducted in accordance with the principles of the Declaration of Helsinki. Participants were recruited from the general population via media advertisements and completed a screening questionnaire that collected general information (sex, height, weight, shift work, prior OSA diagnosis, current OSA treatment, and physical activity levels). The inclusion criteria were as follows: (i) adults aged 18–50 years, (ii) overweight or obese (BMI ≥ 25 < 42 kg/m^2^), (iii) inactive (not participating in an exercise program at baseline), and (iv) diagnosed with mild to moderate SDB (AHI ≥ 5 < 30.0; objective snore ≥10%).

The exercise program was implemented at the Sports Science Laboratory of Reykjavik University. It was developed by the work package leader (J.M.S.), overseen by the PhD student (K.Y.F.), and carried out by MSc students and third-year BSc students in Sports Science. After the initial phases of the study, a postdoctoral researcher (C.J.M.) joined the team to assume coordination responsibilities, working under the supervision of the principal investigator (E.S.A.) and the work package leader (J.M.S.).

### 2.3. Exercise Program

The program was designed based on previous studies and recommendations [35,36,37], seeking the reported benefits [23]. A 12-week program was designed, consisting of three 60 min sessions per week. Each session was divided into the three classic components of a workout: warm-up (between 5 and 10 min depending on the week), main part (between 5 and 10 min depending on the week), and cool-down (between 5 and 10 min depending on the week). The specific duration for each part and each week is detailed in Table 1. The main part was further divided into circuit training (focused on strength endurance development) and brisk walking (focused on aerobic endurance development). This type of training can be considered concurrent exercise, as it combines strength and endurance work within the same session [35]. It should be noted that the program described was designed for inactive individuals. If this program were applied to active people or those with good physical fitness, brisk walking would likely not provide a sufficient stimulus to develop the aerobic component, and it might potentially need to be replaced with running. In addition to the above, since circuit training is focused on strength endurance development, it also has an influence on the aerobic component.

In regard to the circuit training, the number of stations was 10 in weeks one and two, 12 in weeks three and four, 14 from weeks five to eight, and 16 from weeks nine to twelve. The work time at each station was consistently 60 s, with 30 s of rest between stations, except during weeks nine to 12, when the rest period was reduced to 20 s. The exercises were grouped into seven categories [36,38]:

(i) Upper body push:
  − Resistance band chest press. Performed standing with a resistance band anchored behind, pushing forward to activate the chest and triceps.
  − Wall push-up. A beginner-friendly push-up variation performed against the wall to develop upper body strength with minimal load.
  − Dumbbell chest press on a Bosu ball. Lying on a Bosu ball, pressing dumbbells upward, to strengthen the chest and improve core stability.
  − Knee push-up. A modified push-up, performed with knees on the ground to reduce body weight load while training the chest and arms.
  − Triceps dip on a chair. Using a stable chair, lower and raise the body with arms to work the triceps and shoulders.
  − Standard push-up. A full-body push-up from plank position, strengthening chest, shoulders, arms, and core.
(ii) Upper body pull:
  − Renegade row with dumbbells. Performed from a plank position by pulling one dumbbell toward the chest while stabilizing the body with the opposite arm, targeting back, shoulders, and core.
  − Seated resistance band row. Sitting on the floor with legs extended, pull resistance bands toward the torso, engaging the back and biceps.
  − Standing resistance band row. While standing, pull a resistance band anchored in front of the body toward the chest, emphasizing scapular retraction and engaging the upper back and biceps.
  − Bent-over row with resistance band. Performed in a hinged position, pull the band handles toward the waist while keeping the back straight and elbows close, strengthening the upper back and lats.
  − Upright row with resistance band. Standing on the band, pull it upward toward the collarbones with elbows high to target the shoulders and traps.
(iii) Knee-dominant:
  − Resistance band squat walk. Performed in a squat position while taking small lateral steps with a resistance band around the thighs to activate the glutes and quads.
  − Step-up with dumbbells. Involves stepping onto a raised surface with one leg while holding dumbbells, then returning to the starting position to strengthen the quadriceps and glutes.
  − Bosu ball squat. Squat on a Bosu ball to develop balance and lower body strength, focusing on the knees and core.
  − Forward lunge. Step forward and lower the back knee toward the ground, emphasizing knee flexion and muscle engagement in the front leg.
  − Bulgarian split squat. With the rear foot elevated, lower the body into a single-leg squat to target the quads and improve unilateral strength and stability.
(iv) Hip-dominant:
  − Resistance band deadlift. With feet on the band, hinge at the hips to lift against the resistance, engaging glutes and hamstrings.
  − Banded sumo deadlift. A wide-stance deadlift using resistance bands to target the posterior chain, especially the glutes.
  − Glute bridge. Lying on the floor, lifting hips through glute activation, can be performed with both feet planted or with one leg extended to increase difficulty.
  − Single-leg Romanian deadlift with dumbbells. While balancing on one leg, hinge at the hips and lower the dumbbells. Targeting the hamstrings and glutes while enhancing balance, and posterior chain stability.
  − Standing hip extension with band. Push one leg backward against the resistance band to isolate glute activation.
  − Bosu ball hamstring bridge. Lying down with heels on a Bosu ball, lift hips to strengthen the hamstrings and glutes.
(v) Core-specific:
  − Side crunch on Bosu ball. Lying sideways on a Bosu ball, perform crunches to activate the obliques through lateral trunk flexion.
  − Side plank with crunch. From a side plank position, bring the top elbow and knee together to engage the obliques and hip stabilizers.
  − Mountain climber on Bosu. In a plank position with hands on a Bosu ball, alternate driving knees toward the chest to engage the core through dynamic movement.
  − Plank on Bosu ball. Hold a forearm plank with elbows on a Bosu ball to challenge core stability and shoulder control.
  − Medicine ball woodchopper. Perform a rotational movement from high to low with a medicine ball to activate the entire core, especially the obliques.
  − Plank on stability ball. Place forearms on a stability ball while holding a plank position, engaging deep abdominal muscles for balance and control.
(vi) Medicine ball throw:
  − Medicine ball rotational throw. Rotate the torso and explosively throw the medicine ball sideways against a wall to train rotational power.
  − Medicine ball overhead throw. Squat down holding the ball, then extend the hips and arms to throw it vertically overhead, targeting total-body power.
  − Medicine ball forward throw. Raise the ball overhead and throw it straight forward, engaging the core and upper body in an explosive motion.
  − Medicine ball scoop toss. From a squat position, explosively extend and toss the ball upward in a scooping motion to train hip and arm coordination.
(vii) Aerobic-specific exercises:
  − Basic step-up. Step up and down on a platform repeatedly to elevate heart rate and improve lower-body endurance.
  − Jumping jacks. Perform full-body jumping movements by spreading the legs and raising the arms overhead, then returning to the starting position to promote cardiovascular fitness and coordination.
  − High knees. Jog in place while lifting the knees as high as possible to enhance aerobic capacity and coordination.
  − Step jump. Jump onto and off a raised platform in a continuous motion to improve power and cardiovascular fitness.
  − Resisted running with a band. One partner runs forward while the other holds a resistance band to increase sprint effort and conditioning.

It should be noted that the specific exercise chosen within each category was tailored to the characteristics of the participant. For example, when it comes to upper body push exercises, if the participant’s technical level or physical fitness does not allow them to perform standard push-ups at a station, wall push-ups will be performed instead. At the same time, throughout the duration of the program, both the number of stations and the duration have been progressively increased (Table 1) to ensure load progression (see Section 2.4) and allow participants to adapt to the difficulty of the exercises.

The number of exercises in each category is described below: (i) Weeks one and two (ten exercises): one push exercise, two pull exercises, one knee-dominant exercise, three hip-dominant exercises, one specific core exercise, and two specific aerobic exercises. (ii) Weeks three and four (twelve exercises): two push exercises, two pull exercises, two knee-dominant exercises, three hip-dominant exercises, one specific core exercise, and two specific aerobic exercises. (iii) Weeks five to eight (fourteen exercises): two push exercises, two pull exercises, two knee-dominant exercises, three hip-dominant exercises, two specific core exercises, and three specific aerobic exercises. (iv) Weeks nine to twelve (sixteen exercises): two push exercises, two pull exercises, two knee-dominant exercises, four hip-dominant exercises, two specific core exercises, and four specific aerobic exercises.

### 2.4. Planning of Training Volume, Intensity, and Load

The planning adhered to fundamental training principles, particularly the principles of overload, load progression, the relationship between exercise and recovery, and individualization. The principle of overload refers to the need for a stimulus to be sufficiently significant to disrupt equilibrium and trigger adaptation [24]. In the present study, special care was taken to ensure that the training load was adequate, not too high, given that the participants were inactive, but still sufficient to induce adaptations. The principle of load progression refers to the interaction between training volume, intensity, and frequency [25]. In this study, the training load was planned linearly to reach maximum intensity in the final weeks of the program. It is important to note that intensity is a major factor influencing the magnitude of training-induced adaptations in fitness and exercise performance [29]. The principle of the relationship between exercise and recovery states that performance improves not only through the exercise stimulus but also through appropriate post-exercise recovery (supercompensation) [39]. In this study, full recovery between sessions was ensured, considering the characteristics of the participants. Finally, the principle of individualization refers to the need to tailor the training load to each subject’s individual abilities, potential, and learning characteristics [25]. In the present study, within each exercise group, the exercise that best suited the subject’s characteristics was selected.

In this way, the program was planned in advance, detailing the volume, intensity, and total load of each session and week, as well as the specific exercises to be performed. Volume was assessed in terms of real-time exercise duration (not including rest breaks) for each session and week, and intensity was measured using the Borg RPE [31]. The RPE scale ranges from 6 to 20 and is used to assess how hard a person feels they are working during physical activity or exercise. Participants were instructed prior to the start of the exercise sessions on how the 6 to 20 RPE scale works and what it represents, both verbally and using a visual chart illustrating exercise intensity. To integrate volume and intensity into a single value known as training impulse (TRIMP) [40], also referred to as training load, volume (actual minutes of exercise, excluding rest periods) was multiplied by intensity (RPE) [33], resulting in arbitrary units (Table 1). At the end of each session, participants reported their RPE for the three parts of the training: warm-up, main part (circuit training and brisk walking), and cool-down. In order to monitor whether the planned intensity matched the real intensity of the program.

### 2.5. Statistical Analysis

A comparison of the mean values for training intensity, and total load was carried out between the planned sessions and what was actually performed, based on the RPE reported by each participant at the end of each session in the program. To determine whether the observed differences were statistically significant, a paired t-test was performed, calculating the *t*-value, *p*-value, and confidence intervals. Similarly, the effect sizes of the differences were calculated using Cohen’s d [41]. The level of statistical significance was set at *p* < 0.05 for all analyses. All statistical calculations were performed using IBM SPSS Statistics, version 25.

## 3. Results

**Figure 1:**
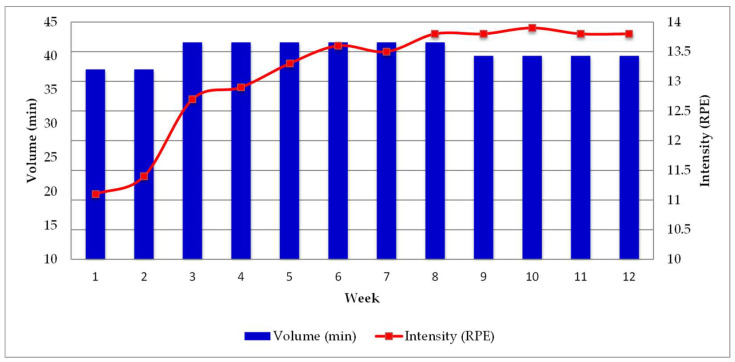
Progression of volume and intensity week by week.

Table 2 shows the differences between planned and performed training intensity and training load. Differences between planned and performed volume are not presented because volume was identical across all sessions and weeks. Overall, the performed intensity [t = 1.501; 95%CI (−0.318, 0.551); *p* = 0.591; ES = 0.17] and training load [t = 0.728; 95%CI (−11.570, 23.004); *p* = 0.482; ES = 0.21] did not show statistically significant differences, although in percentage terms, the values were slightly higher than planned (by 3.2% and 2.5%, intensity and training load, respectively). By weeks, no statistically significant differences were found between performed and planned intensity and training load in weeks 1–2 (*p* = 0.090; *p* = 0.098 for intensity and training load, respectively), weeks 3–4 (*p* = 0.090; *p* = 0.081 for intensity and training load, respectively), and weeks 5–8 (*p* = 0.245; *p* = 0.181 for intensity and training load, respectively). However, in percentage terms, there were deviations between the planned and performed values (10.8%, 5.8%, and 1.5%, weeks 1–2, 3–6, and 5–8, respectively). In contrast, statistically significant differences were found in weeks 9–12 for both intensity [*t* = 27.000; 95% CI (–0.755, –0.505); *p* < 0.001; ES = −13.5] and training load [*t* = –26.001; 95% CI (–29.182, –22.818); *p* < 0.001; ES = –13]. In percentage terms, the difference was −4.8% in intensity and −4.9% in training load. Regarding each content block, the performed intensity for brisk walking was lower than planned (12.5 vs. 11.7; −6.4%), whereas it was higher for the warm-up (12.9 vs. 7; 4.5%) and the cool-down (7 vs. 9; 28.6%). Figure 1 shows the weekly progression of volume and intensity performed.

## 4. Discussion

The objectives of this study were (i) to design in detail an exercise program for individuals with sleep-disordered breathing that would be reproducible, and (ii) to present a system for monitoring training load (volume and intensity) within such a program. The protocol presented is described in detail, which would allow for reproducibility. At the same time, it has been shown to be effective in improving OSA severity (AHI) as well as skeletal muscle mass, physical fitness, and HRQoL [16]. Furthermore, to the best of the authors’ knowledge, this is the first study to carry out a thorough monitoring of training volume, intensity, and training load, also revealing that, overall, there is consistency between the planned and the performed training.

One of the most important aspects of scientific studies is their potential to be replicated, that is, their reproducibility [42]. The training process, whether applied to healthy individuals or patients with disease is complex, and this complexity can sometimes hinder the reproducibility of the study. Such difficulty can complicate the execution of research and, in some cases, represent a methodological weakness that may even call into question the validity of the conclusions. In this regard, the Consensus on Exercise Reporting Template (CERT) outlines 16 items designed to fully describe exercise interventions in clinical trials and evaluative studies, directly addressing the issue of insufficient detail in the reporting of exercise programs [43]. Using this tool, it has been reported that 52% of 23 exercise-based intervention studies in patients with type 2 diabetes mellitus could not be replicated [44]. Similarly, a systematic review of exercise interventions in solid organ transplant recipients concluded that many RCTs presented difficulties in replication due to poor reporting [45]. To the best of the authors’ knowledge, no studies have evaluated the reporting quality and reproducibility of studies that use exercise interventions to improve various sleep-related parameters. However, there is literature supporting the need for standardization in this area [46].

If we focus on the CERT items directly related to the exercise intervention, such as progression (rules and description) and the detailed description of the exercise program [43], one work that analyzed 28 studies including exercise interventions in patients with various diseases concluded that only 15% and 23% of the studies reported the progression rules and description, respectively, and just 24% adequately described the exercise program itself [47]. The present study not only provided a detailed description of the exercise program to be performed (see Section 2.3), aimed at improving AHI (among other parameters), but also thoroughly outlined both the rules and the description of progression in training volume, intensity, and load (Table 1). This detailed description facilitates reproducibility. Regarding the program detail, previous studies focused on SDB have sometimes combined highly diverse aerobic components, such as Nordic walking, aquagym, and gymnastics [15] or elliptical and bicycle ergometer training [48]. This lack of specificity is even more relevant in strength or strength–endurance programs, where at best, only the muscle group targeted is mentioned [17,18,22], or, in the best-case scenario, the exercise name is listed without any explanation [48]. With respect to load progression, some studies do not report any progression at all [14,15], while others describe only minimal progression in volume (e.g., time or number of sessions) [18] or in initial training intensity [19]. In any case, the progressions are described in a very superficial manner.

When analyzing the planned and actual training volume, intensity, and load, it is important to highlight the need to quantify these parameters. In fact, if they are not quantified with precise, thorough, and in-depth information, the findings of a training study hold very little or no value [49]. In the present study, exercise intensity was assessed using the RPE, in line with previous studies [17,19]. This method was chosen because it allowed the integration of both circuit training (strength endurance exercise) and brisk walking (endurance exercise) into a single measurement approach. This contrasts with other studies that determine repetitions based on one-repetition maximum testing for strength exercises [22], or use heart rate [21] or anaerobic threshold [17] measures for endurance training. Nevertheless, RPE may be influenced by factors such as pre-exercise fatigue [50], sleep deprivation [51], or mood state [52]. Similarly, the present study is the first to quantify training intensity (volume × intensity) in an exercise program aimed at individuals with SDB. In addition to this quantification, it allowed confirmation (after the completion of the exercise) that the program had been followed since there were no significant differences in either intensity (*p* = 0.591) or training load (*p* = 0.482), although there were only minor deviations between the planned and performed intensity and training load, with the performed values being slightly higher than planned (by 3.2% and 2.5%, respectively) (Table 2). There were variations throughout the weeks, with a non-significant underestimation observed in weeks 1–2 (intensity: 10.8%; training load: 11.1%; *p* = 0.090; *p* = 0.098, respectively) and an overestimation in weeks 9–12 (−4.8% for intensity and −4.9% for training load, respectively; *p* < 0.001). Nevertheless, the most notable finding was the underestimation of intensity during the warm-up and cool-down phases (4.6% and 28.6%, respectively), suggesting that the intensity of these segments may need to be reconsidered, at least for inactive individuals such as those included in the present study.

This study has several limitations. First, although the exercise program has been described in detail, the protocol is specifically designed for inactive individuals with SDB and therefore cannot be generalized to other populations, though it can be adapted. Second, while the protocol provides a clear description of the internal training load, it is an individual perception that may differ from one participant to another. Third, if objective measures such as heart rate had been used to assess intensity, they could have provided complementary information to the use of RPE. Fourth, both the perceived intensity and adherence to the exercise program could be influenced by gender and BMI.

## 5. Conclusions

The conclusions of this study were as follows: (i) a detailed exercise program was described for individuals with sleep-disordered breathing, which is reproducible in terms of content, as well as training volume, intensity, and load; (ii) the RPE proved to be a valid parameter for quantifying intensity, allowing integration of all parts of the session (warm-up, main part, and cool-down), as well as content of various types, such as circuit training (strength endurance) and brisk walking (aerobic endurance); (iii) quantifying the training load enabled proper planning and adaptation in accordance with the principles of training; (iv) the planned and performed programs (quantified via participants’ RPE) matched appropriately since there were no significant differences in either intensity or training load, although in percentage terms there was a minimal difference (3.2% and 2.5%, respectively). Thus, this program can be reproduced and applied to this type of participants.

## Figures and Tables

**Table 1:** Planned values for real exercise volume (excluding rest periods) in minutes, intensity (RPE), and training load (arbitrary units—UA).

	Volume (min)					Intensity (RPE)					Training Load (Volume × Intensity) (AU)
Week	WU	CC	BW	CD	Total	WU	CC	BW	CD	Total	
1–2	10	10	8	10	38	8	14	12	7	10.2	386.0
3–4	10	12	10	10	42	9	15	12	7	12.1	508.4
5–8	8	14	12	8	42	9	16	13	7	13.4	561.5
9–12	5	16	14	5	40	9	18	13	7	14.5	579.0

WU, warm-up; CC, circuit training; BW, brisk walking; CD, cool-down.

**Table 2:** Planned and performed training intensity and load (TL), and percentage differences between them.

	Planned Intensity (RPE)					Performed Intensity (RPE)					Diff. (%)	Planned TL (UA)	Performed TL (UA)	Diff. (%)
Week	WP	CC	BW	CD	Total	WP	CC	BW	CD	Total				
1–2	8	14	12	7	10.2	9.4	15.3	10.9	9.5	11.3	10.8	386.0	428.9	11.1
3–4	9	15	12	7	12.1	9.2	15.3	11.9	9.1	12.8	5.8	508.4	536.5	5.5
5–8	9	16	13	7	13.4	9.3	15.9	12.4	8.8	13.6	1.5	561.5	570.4	1.6
9–12	9	18	13	7	14.5	9.1	16.8	11.8	8.7	13.8	−4.8	579.0	550.4	−4.9
Mean	8.8	15.8	12.5	7	12.5	9.2	15.8	11.7	9.0	12.9	3.2	508.7	521.5	2.5

TL, training load; Diff., differences; WU, warm-up; CC, circuit training; BW, brisk walking; CD, cool-down.

## Data Availability

Data are contained within the article.

## Abbreviations

The following abbreviations are used in this manuscript:

AHI	Apnea–Hypopnea Index
BMI	Body Mass Index
CERT	Consensus on Exercise Reporting Template
CI	Confidence interval
ES	Effect sizes
HR	Heart rate
HRQoL	Health-Related Quality of Life
OSA	Obstructive Sleep Apnea
RCT	Randomized Controlled Trial
RPE	Rating of Perceived Exertion
SDB	Sleep-Disordered Breathing
TRIMP	Training Impulse

## References

[1] Lobelo F., Stoutenberg M., Hutber A. (2014). The Exercise is Medicine Global Health Initiative: A 2014 update. Br. J. Sports Med..

[2] World Health Organization (2020). WHO Guidelines on Physical Activity and Sedentary Behaviour.

[3] Bull F.C., Al-Ansari S.S., Biddle S., Borodulin K., Buman M.P., Cardon G., Carty C., Chaput J.-P., Chastin S., Chou R. (2020). World Health Organization 2020 guidelines on physical activity and sedentary behaviour. Br. J. Sports Med..

[4] Kraus W., Powell K., Haskell W., Janz K., Campbell W., Jakicic J., Troiano R., Sprow K., Torres A., Piercy K.L. (2019). Physical activity, all-cause and cardiovascular mortality, and cardiovascular disease. Med. Sci. Sports Exerc..

[5] Pescatello L.S., Buchner D.M., Jakicic J.M., Powell K.E., Kraus W.E., Bloodgood B., Campbell W.W., Dietz S., Dipietro L., George S.M. (2019). Physical activity to prevent and treat hypertension: A systematic review. Med. Sci. Sports Exerc..

[6] Smith A.D., Crippa A., Woodcock J., Brage S. (2016). Physical activity and incident type 2 diabetes mellitus: A systematic review and dose–response meta-analysis of prospective cohort studies. Diabetologia.

[7] McTiernan A., Friedenreich C.M., Katzmarzyk P.T., Powell K.E., Macko R., Buchner D., Pescatello L.S., Bloodgood B., Tennant B., Vaux-Bjerke A. (2019). Physical activity in cancer prevention and survival: A systematic review. Med. Sci. Sports Exerc..

[8] Núñez-Cortés R., Salazar-Méndez J., Nijs J. (2023). Physical activity as a central pillar of lifestyle modification in the management of chronic musculoskeletal pain: A narrative review. J. Funct. Morphol. Kinesiol..

[9] Singh B., Olds T., Curtis R., Dumuid D., Virgara R., Watson A., Szeto K., O’Connor E., Ferguson T., Eglitis E. (2023). Effectiveness of physical activity interventions for improving depression, anxiety and distress: An overview of systematic reviews. Br. J. Sports Med..

[10] Ferreira J.P., Gregson J., Duarte K., Gueyffier F., Rossignol P., Zannad F., Pocock S. (2018). Individualizing treatment choices in the systolic blood pressure intervention trial. J. Hypertens..

[11] Peng J., Yuan Y., Zhao Y., Ren H. (2022). Effects of exercise on patients with obstructive sleep apnea: A systematic review and meta-analysis. Int. J. Environ. Res. Public Health.

[12] Randerath W., de Lange J., Hedner J., Ho J.P.T.F., Marklund M., Schiza S., Steier J., Verbraecken J. (2022). Current and novel treatment options for obstructive sleep apnoea. ERJ Open Res..

[13] Randerath W., Verbraecken J., de Raaff C.A.L., Hedner J., Herkenrath S., Hohenhorst W., Jakob T., Marrone O., Marklund M., McNicholas W.T. (2021). European respiratory society guideline on non-CPAP therapies for obstructive sleep apnoea. Eur. Respir. Rev..

[14] Araújo C.E.L., Ferreira-Silva R., Gara E.M., Goya T.T., Guerra R.S., Matheus L., Toschi-Dias E., Rodrigues A.G., Barbosa E.R.F., Fazan R. (2021). Effects of exercise training on autonomic modulation and mood symptoms in patients with obstructive sleep apnea. Braz. J. Med. Biol. Res..

[15] Berger M., Barthélémy J.-C., Hupin D., Raffin J., Dupré C., Labeix P., Costes F., Gaspoz J.-M., Roche F. (2018). Benefits of supervised community physical activity in obstructive sleep apnoea. Eur. Respir. J..

[16] Fridgeirsdottir K.Y., Murphy C.J., Islind A.S., Árnadóttir B.S., Hrubos Strøm H., Arnardottir E.S., Saavedra J.M. (2025). Effects of exercise and a lifestyle app on sleep disordered breathing, physical health, and quality of life. ERJ Open Res..

[17] Goya T.T., Ferreira-Silva R., Macedo Gara E., Guerra R.S., Barbosa E.R.F., Toschi-Dias E., Cunha P.J., Negrão C.E., Lorenzi-Filho G., Ueno-Pardi L.M. (2021). Exercise training reduces sympathetic nerve activity and improves executive performance in individuals with obstructive sleep apnea. Clinics.

[18] Guerra R.S., Goya T.T., Silva R.F., Lima M.F., Barbosa E.R.F., Alves M.J.D.N.N., Rodrigues A.G., Lorenzi-Filho G., Negrão C.E., Ueno-Pardi L.M. (2019). Exercise training increases metaboreflex control in patients with obstructive sleep apnea. Med. Sci. Sports Exerc..

[19] Jurado-García A., Molina-Recio G., Feu-Collado N., Palomares-Muriana A., Gómez-González A.M., Márquez-Pérez F.L., Jurado-Gamez B. (2020). Effect of a graduated walking program on the severity of obstructive sleep apnea syndrome: A randomized clinical trial. Int. J. Environ. Res. Public Health.

[20] Lins-Filho O., Germano-Soares A.H., Aguiar J.L.P., de Almedia J.R.V., Felinto E.C., Lyra M.J., Leite D.B., Moura M.A.S., Kline C.E., Pedrosa R.P. (2023). Effect of high-intensity interval training on obstructive sleep apnea severity: A randomized controlled trial. Sleep Med..

[21] Lins-Filho O.L., Pedrosa R.P., Gomes J.M.L., Moraes S.L.D., Vasconcelos B.C.E., Lemos C.A.A., Pellizzer E.P. (2020). Effect of exercise training on subjective parameters in patients with obstructive sleep apnea: A systematic review and meta-analysis. Sleep Med..

[22] Servantes D.M., Javaheri S., Kravchychyn A.C.P., Storti L.J., Almeida D.R., de Mello M.T., Cintra F.D., Tufik S., Bittencourt L. (2018). Effects of exercise training and CPAP in patients with heart failure and OSA: A preliminary study. Chest.

[23] Giannaki C.D., Sakkas G.K., Hadjigeorgiou G.M., Manconi M., Bargiotas P. (2024). Unfolding the role of exercise in the management of sleep disorders. Eur. J. Appl. Physiol..

[24] Lambert M.I., Viljoen W., Bosch A.N., Pearce A.J., Sayers M., Schwellnus M.P. (2008). General principles of training. Olympic Textbook of Medicine in Sport.

[25] Bompa T.O., Buzzichelli C. (2019). Periodization: Theory and Methodology of Training.

[26] Garber C.E., Blissmer B., Deschenes M.R., Franklin B.A., Lamonte M.J., Lee I.-M., Nieman D.C., Swain D.P. (2011). Quantity and quality of exercise for developing and maintaining cardiorespiratory, musculoskeletal, and neuromotor fitness in apparently healthy adults: Guidance for prescribing exercise. Med. Sci. Sports Exerc..

[27] Wollesen B., Herden M., Lamberti N., Giannaki C.D. (2024). Defining and reporting exercise intensity in interventions for older adults: A modified Delphi process. Eur. Rev. Aging Phys. Act..

[28] Papale O., Festino E., Di Rocco F., Foster C., Prestanati I., Serafini S., Izzicupo P., Cortis C., Fusco A. (2023). The impact of a multidimensional physical activity intervention on glycemic control in type 1 diabetes: A preliminary study. J. Funct. Morphol. Kinesiol..

[29] Mujika I. (2010). Intense training: The key to optimal performance before and during the taper. Scand. J. Med. Sci. Sports.

[30] Halson S.L. (2014). Monitoring training load to understand fatigue in athletes. Sports Med..

[31] Borg G.A.V. (1962). Physical Performance and Perceived Exertion.

[32] Bordoli C., Varley I., Sharpe G.R., Johnson M.A., Hennis P.J. (2023). Effects of oral lactate supplementation on acid–base balance and prolonged high-intensity interval cycling performance. J. Funct. Morphol. Kinesiol..

[33] Foster C., Florhaug J.A., Franklin J., Gottschall L., Hrovatin L.A., Parker S., Doleshall P., Dodge C. (2001). A new approach to monitoring exercise training. J. Strength Cond. Res..

[34] Milani J.G.P.O., Milani M., Verboven K., Cipriano G., Hansen D. (2024). Exercise intensity prescription in cardiovascular rehabilitation: Bridging the gap between best evidence and clinical practice. Front. Cardiovasc. Med..

[35] Agner V.F.C., Garcia M.C., Taffarel A.A., Mourão C.B., da Silva I.P., da Silva S.P., Peccin M.S., Lombardi I. (2018). Effects of concurrent training on muscle strength in older adults with metabolic syndrome: A randomized controlled clinical trial. Arch. Gerontol. Geriatr..

[36] Saavedra J.M., Kristjánsdóttir H., Gunnarsson S.B., García Hermoso A. (2021). Effects of 2 physical exercise programs (circuit training and brisk walk) carried out during working hours on multidimensional components of workers’ health: A pilot study. Int. J. Occup. Med. Environ. Health.

[37] Timmons J.F., Minnock D., Hone M., Cogan K.E., Murphy J.C., Egan B. (2018). Comparison of time-matched aerobic, resistance, or concurrent exercise training in older adults. Scand. J. Med. Sci. Sports.

[38] Lawrence D., Hope S. (2008). Advanced Circuit Training: A Complete Guide to Progressive Planning for Fitness Training.

[39] Barnett A. (2006). Using recovery modalities between training sessions in elite athletes: Does it help?. Sports Med..

[40] Banister E.W., Calvert T.W., Savage M.V., Bach T. (1975). A systems model of training for athletic performance. Aust. J. Sports Med..

[41] Cohen J. (1988). Statistical Power Analysis for the Behavioral Sciences.

[42] Ioannidis J.P.A., Greenland S., Hatlay M.A., Khoury M.J., Macleod M.R., Moher D., Schulz K.F., Tibshirani R. (2014). Increasing value and reducing waste in research design, conduct, and analysis. Lancet.

[43] Slade S.C., Finnegan S., Dionne C.E., Underwood M., Buchbinder R. (2018). The Consensus on Exercise Reporting Template (CERT) applied to exercise interventions in musculoskeletal trials demonstrated good rater agreement and incomplete reporting. J. Clin. Epidemiol..

[44] Hacke C., Schreiber J., Weisser B. (2022). Application of the Templates TIDieR and CERT reveal incomplete reporting and poor replicability of exercise interventions for type 2 diabetes mellitus. Curr. Diabetes Rev..

[45] Raje U., Saumur T.M., Pesce de Souza F., Mathur S., Janaudis-Ferreira T. (2021). Quality of the reporting of exercise interventions in solid organ transplant recipients: A systematic review. McGill J. Med..

[46] Zhang F., Wang H., Huang L., Bai Y., Wang W., Zhang H. (2023). Effect of exercise interventions for sleep quality in patients. A systematic review and meta-analysis. Int. Urol. Nephrol..

[47] Hansford H.J., Wewege M.A., Cashin A.G., Hagstrom A.D., Clifford B.K., McAuley J.H., Jones M.D. (2022). If exercise is medicine, why don’t we know the dose? An overview of systematic reviews assessing reporting quality of exercise interventions in health and disease. Br. J. Sports Med..

[48] Kline C.E., Crowley E.P., Ewing G.B., Burch J.B., Blair S.N., Dunsire J.L., Davis J.M., Youngstedt S.D. (2011). The effect of exercise training on obstructive sleep apnea and sleep quality: A randomized controlled trial. Sleep.

[49] Mujika I. (2013). The alphabet of sport science research starts with Q. Int. J. Sports Physiol. Perform..

[50] Gil-Moreno G., Palmi J., Prat-Subirana J.A. (2017). Assessment of the subjective perception of fatigue in competition motorcyclists Rally-Raid Dakar. Accion Psicol..

[51] Kong Y., Yu B., Guan G., Wang Y., He H. (2025). Effects of sleep deprivation on sports performance and perceived exertion in athletes and non-athletes: A systematic review and meta-analysis. Front. Physiol..

[52] Viana B.F., Pires F.O., Inoue A., Micklewright D., Santos T.M. (2016). Correlates of mood and RPE during multi-lap off-road cycling. Appl. Psychophysiol. Biofeedback.

